# Genomic instability in a chronic lymphocytic leukemia patient with mono-allelic deletion of the *DLEU* and *RB1* genes

**DOI:** 10.1186/s13039-019-0417-5

**Published:** 2019-01-31

**Authors:** María Paulina Nava-Rodríguez, Martín Daniel Domínguez-Cruz, Lilia Beatriz Aguilar-López, César Borjas-Gutiérrez, María Teresa Magaña-Torres, Juan Ramón González-García

**Affiliations:** 1 Doctorado en Genética Humana, Centro Universitario de Ciencias de la Salud. Universidad de Guadalajara, Guadalajara, Jalisco Mexico; 2 División de Genética, Centro de investigación Biomédica de Occidente, Instituto Mexicano del Seguro Social, CIBO-IMSS, Guadalajara, Jalisco Mexico; 3 Instituto Jalisciense de Ciencias Forenses, Secretaría de Salud Jalisco, Guadalajara, Jalisco México; 4 UMAE H. Especialidades-CMNO, Instituto Mexicano del Seguro Social, Guadalajara, Jalisco México

**Keywords:** Chronic lymphocytic leukemia, 13q14 deletion, *DLEU* and *RB1* gene loss, Genomic instability

## Abstract

**Background:**

The most frequent cytogenetic abnormality detected in chronic lymphocytic leukemia (CLL) patients is the presence of a deletion within the chromosome band 13q14. Deletions can be heterogeneous in size, generally encompassing the *DLEU1* and *DLEU2* genes (minimal deleted region), but at times also including the *RB1* gene. The latter, larger type of deletions are associated with worse prognosis.

Genomic instability is a characteristic of most cancers and it has been observed in CLL patients mainly associated with telomere shortening.

**Case presentation:**

Cytogenetic and fluorescence in situ hybridization studies of a CLL patient showed a chromosomal translocation t(12;13)(q15;q14), a mono-allelic 13q14 deletion encompassing both the *DLEU* and *RB1* genes, and genomic instability manifested as chromosomal breaks, telomeric associations, binucleated cells, nucleoplasmic bridges, and micronucleated cells.

In conclusion, our CLL patient showed genomic instability in conjunction with a 13q14 deletion of approximately 2.6 megabase pair involving the *DLEU* and *RB1* genes, as well as other genes with potential for producing genomic instability due to haploinsufficiency.

## Background

Chronic lymphocytic leukemia (CLL) is a B-cell lymphoproliferative disorder commonly affecting elderly people [1]. The most frequent cytogenetic abnormality detected by interphase fluorescence in situ hybridization (FISH) is the presence of a deletion within the chromosome band 13q14. Deletions can be heterogeneous in size, generally encompassing the *DLEU1* and *DLEU2* genes (minimal deleted region), but at times also including the *RB1* gene. The latter, larger type of deletions are associated with worse prognosis [1–3].

Genomic instability is present in most cancers. It is characterized by a high frequency of mutations occurring within the cell genome. Alterations in several pathways involved in detecting and repairing DNA damage, telomere maintenance, and chromosomal mitotic segregation will cause increased frequencies of base pair mutation, microsatellite instability, telomere shortening, and chromosome instability mainly manifested as numerical and structural chromosomal abnormalities, micronuclei, and nucleoplasmic bridges (NPB) [4–8]. Several forms of genomic instability has been observed in CLL patients [9–14]. We here report a CLL patient with genomic instability and a large mono-allelic 13q14 deletion encompassing the *DLEU1*, *DLEU2* and *RB1* genes.

## Case presentation

A 61-year-old male patient with bilateral adenomegaly in the neck showed in his peripheral blood a leukocyte count of 49.1 X10^9^/L, with 90% of lymphocytes. Immunophenotyped cells were positive for CD20, CD5, and CD23 surface antigens; therefore, after being diagnosed with CLL (Rai IV), the hematologist administered chemotherapy consisting of cyclophosphamide, adriamycin, vincristine, and prednisone, but the patient’s disease was refractory to such treatment. Next, the patient was started on fludarabicin and rituximab but an adverse reaction was later reported. Another cycle of treatment with cyclophosphamide and prednisone was administered with no response since leukocytosis remained during the 3 years that preceded his demise.

### Cytogenetic studies

Peripheral blood lymphocytes obtained before therapy were cultured in RPMI-1640 medium and stimulated with a mixture of phorbol-12-myristate-13-acetate plus pokeweed mitogen at concentrations previously described [15, 16]. After 72 h of incubation, metaphase cells were obtained from cell cultures harvested by standard methods. Chromosomes were stained following the Giemsa-trypsin banding protocol and analyzed under the microscope. Results were interpreted following the ISCN (2016) recommendations [17].

### FISH studies

Three fluorescent in situ hybridization (FISH) analyses were performed separately. In a first analysis, we used a mixture of the dual color 13q14.3-deletion probe (Cytocell, LPH 006), which covers the *DLEU1* and *DLEU2* genes *(*labeled in red) and the 13q subtelomere sequence (labeled in green), plus the RB1 (13q14) probe (labeled in green; Kreatech, KI-40001). According to information published by the providers, the red labeled probe targeted to the *DLEU* genes is conformed of two separated fragments of 215 and 93 kb, which together span a sequence from chr13:49962705 to 50,671,242 (hg38; ~ 700 kb). As for the RB1 (13q14) probe, it covers a continuous sequence approximately from chr13:48062708 to 48,801,516 (hg38; ~ 740 kb). A second FISH examination was performed using the *MDM2* Amplification probe (Cytocell, LPS 016). We also performed a third FISH study with the dual color *P53/ATM* probe (Cytocell, LPH 052). In all these FISH studies, cells were counterstained with 4′,6-diamino-2-phenylindole.

## Results

**Fig. 1:**
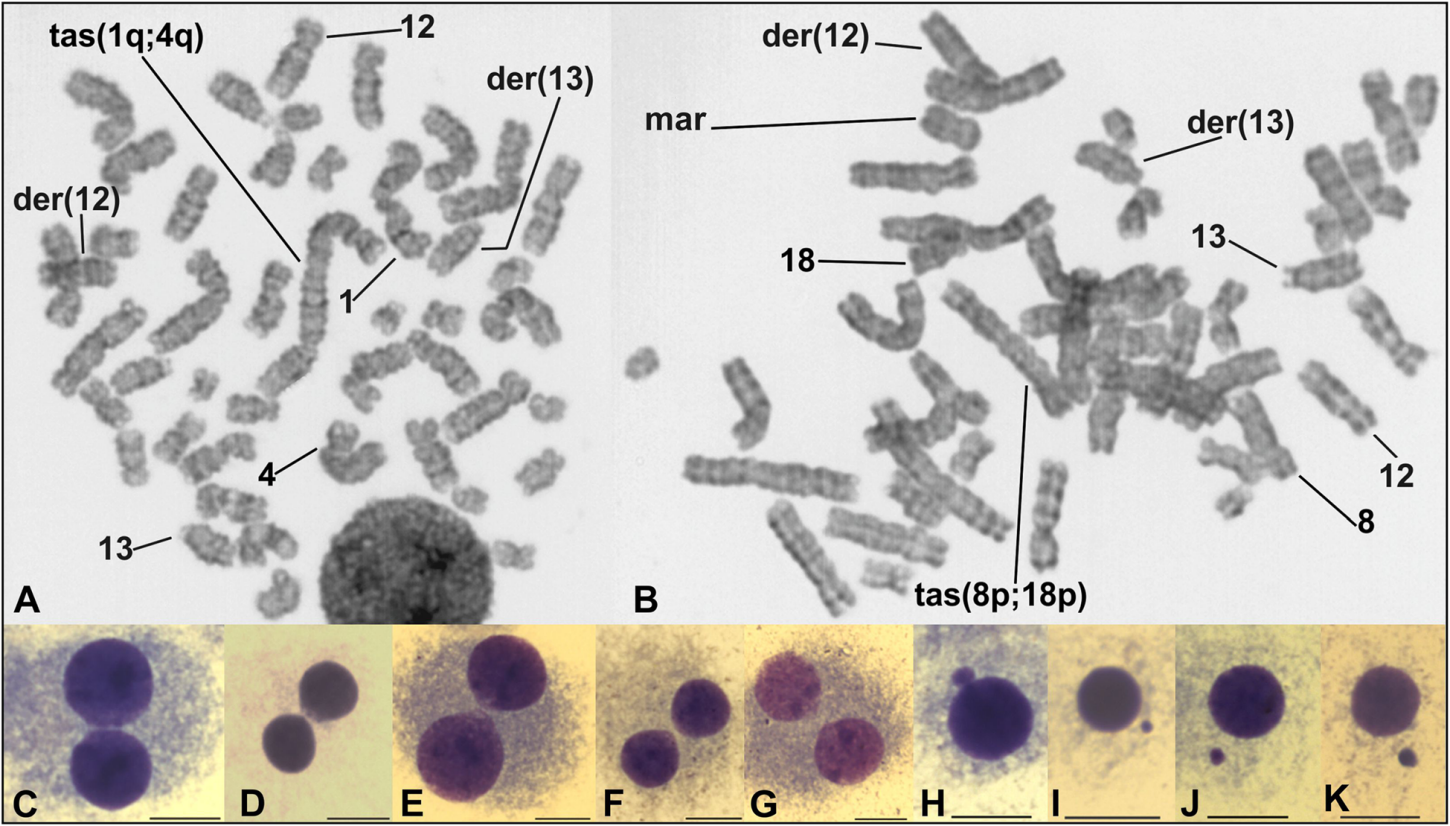
Cells observed at GTG-banding analysis. **a** and **b** Metaphase cells displaying the translocation t(12;13)(q15;q14). Telomeric associations (tas) were also observed in both cells. Moreover, a marker chromosome is shown in **b**. **c**-**g** Binucleated cells showing NPB. **h**-**k** Micronucleated cells. Scaling bar = 10 μm

The Giemsa-trypsin banded metaphase analysis displayed the karyotype 46,XY,t(12;13)(q15;q14)[25]/46,XY[2] (Fig. 1 a and b). Seven out of these 25 cells carrying the translocation t(12;13) showed other single-cell abnormalities as chromosomal breaks, translocations, marker chromosomes, and telomeric associations (Fig. 1 a-b). In addition, while performing the chromosomal banding analysis we observed micronucleated and binucleated cells (Fig. 1 c-k). Micronuclei were observed in 68 out of the 1434 scored cells (4.7%), which is within the range of 2.23 to 4.8% of basal micronucleus frequency reported by Hamurcu et al. in six CLL patients [13]. Moreover, thirty out of the 1434 scored cells (2.1%) were binucleated cells; and, eleven of them (0.77%) displayed NPB (Fig. 1 c-g), which is statistically different (*p* < 0.001; Fisher’s exact test) from the overall baseline NPB frequency reported by Cai et al. in the peripheral blood lymphocytes of 121 healthy individuals from the general population (0.46 ± 0.20 per 1000 binucleated cells) [18].

**Fig. 2:**
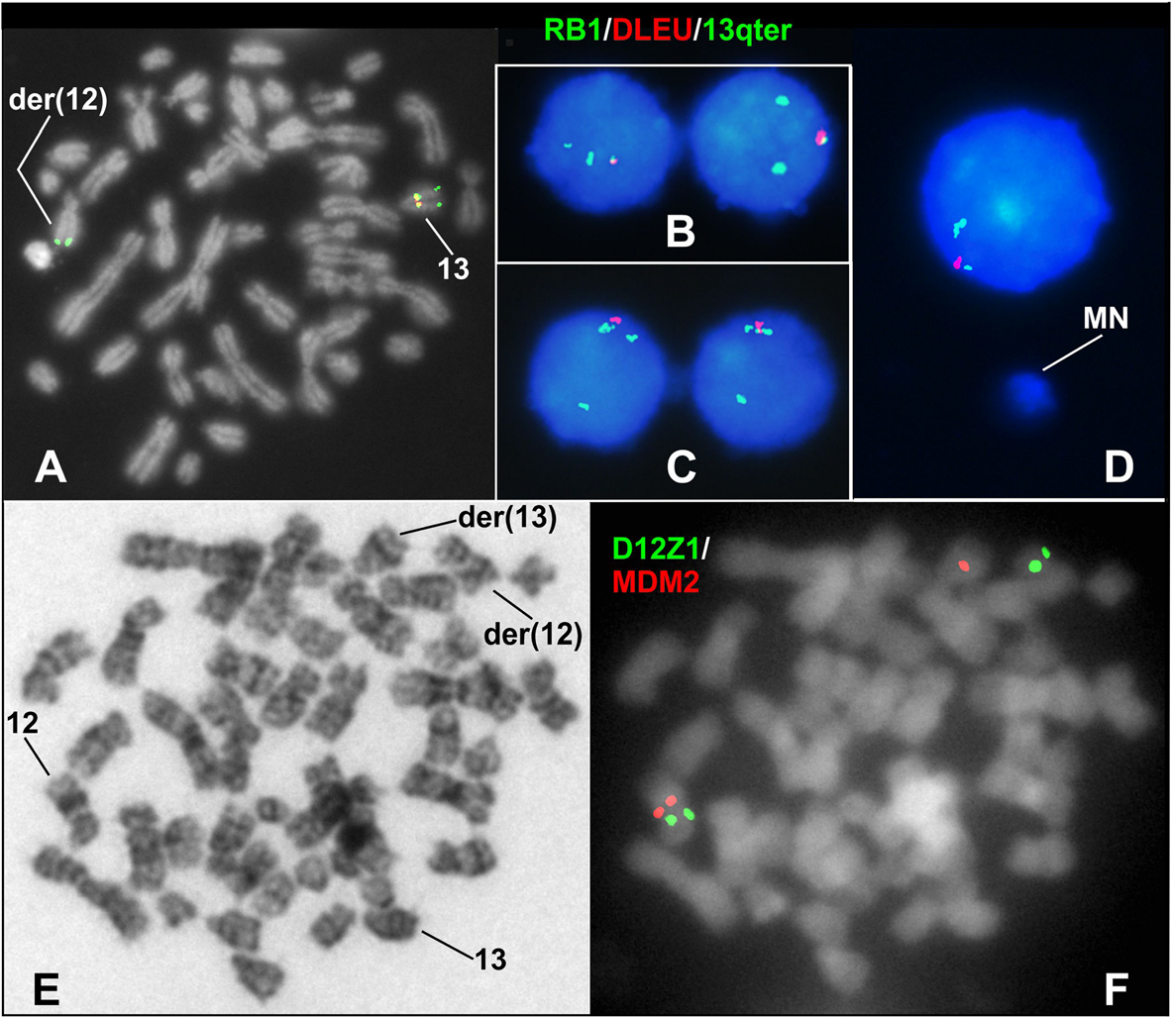
FISH observations. **a**-**d** FISH study performed with a mixture of DLEU, 13q subtelomere, and RB1 probes. In **a**, the normal chromosome 13 shows the three expected signals (*RB1* (green), *DLEU* (red) and 13q subtelomere (green)), whereas, only one 13q subtelomere signal is observed on the der(12) chromosome. All analyzed binucleated cells with NPB (**b** and **c**), as well as micronucleated cells (**d**), displayed a signal pattern concordant with *RB1-DLEU* deletion. **e** GTG-banded metaphase with translocation t(12;13). **f** The same metaphase was studied by FISH with the *MDM2* amplification probe. The *MDM2* red signal is observed on the der(13) chromosome evidencing that the breakpoint on the der(12) occurred centromeric to the *MDM2* gene. MN = micronucleus

The FISH study with the mixture of DLEU, 13q subtelomere, and RB1 probes aimed to reveal the status of the *DLEU* and *RB1* genes in the derivative chromosomes of the translocation. We found a heterozygous 13q14 deletion of approximately 2.6 megabase pair (nearby from chr13:48062708 to 50,671,242, (hg38)), which included both the *DLEU* and *RB1* genes (Fig. 2 a). Such a deletion was observed in 92% of the 200 scored nuclei. Strikingly, all binucleated cells, as well as cells having micronuclei, analyzed in this FISH experiment, were positive for that deletion (Fig. 2 b-d). Subsequent FISH analysis with the *MDM2* probe was done in order to explore the location of the 12q breakpoint in the t(12;13) translocation; such a breakpoint was located centromeric to the *MDM2* gene (Fig. 2 e-f). In addition, no evidence of trisomy for chromosome 12 was found after analyzing 200 interphase cells, which consistently showed two *MDM2* and two *D12Z1* probe signals. Furthermore, FISH study with the dual color *P53/ATM* probe for searching deletions of these genes disclosed normal results in the 200 scored nuclei (not shown).

## Discussion and conclusion

The translocation t(12;13)(q15;q14) observed in our patient caused a heterozygous deletion of the *DLEU* and *RB1* genes. There are other translocations registered in the Mitelman Database of Chromosome Aberrations and Gene Fusions in Cancer [19] that affect the 13q14 chromosomal band. However, there are only four cases of translocation t(12;13) sharing the breakpoints observed in our case [20–23]. FISH analysis in two of these four cases showed 13q14 deletions of D13S319 or D13S25 markers, which are within sequence of the *DLEU1* and *DLEU2* genes [22, 23]. The 13q14.3 chromosomal band is the minimal deleted region for B-cell CLL and it is known for its potential tumor-suppressing function. It contains various tumor suppressor gene candidates, mainly the *DLEU1* and *DLEU2* genes whose loss has been considered as an early step in the development of the disease [24, 25].

It is hard to explain the genomic instability present in our patient since there are several mechanisms that could be related with its presence, as telomere shortening suggested by the presence of telomeric associations, as well as the gene content of the 13q14 region. There are several genes mapping in the deleted segment observed in our patient (chr13:48062708 to 50671242, hg38) whose haploinsufficiency has the potential for causing genome instability. The *Mir-16* gene is involved in the DNA damage signaling pathway [26]; the *RCBTB2* and *SETDB2* genes play a role in chromosome condensation and segregation during mitosis [27, 28]; and, the *KPNA3* gene is involved in the nuclear import of protein MeCP2 that has important roles in regulating chromatin structure [29]. Regarding the *RB1* gene, its protein plays a well-known role in G1 to S phase progression. Alteration of this mechanism of regulation causes a replicative stress, resulting in the production of DNA double-stranded breaks [30]. Interestingly, new roles of the RB1 protein have been recently identified and all of them are directly involved in the maintenance of genome stability [31, 32]. Coschi et al. [31] linked haploinsufficiency of the *RB1* gene with a wide variety of aberrant processes affecting the normal cell cycle as alteration of the number of centrosomes, defects in the mitotic spindle assembly, occurrence of merotelic kinetochore attachments, and failure of cytokinesis generating either binucleated cells or NPB. Therefore, from this perspective, genomic instability could be a variable phenotypic consequence comparable to that occurring in contiguous gene syndromes, where deletions have variable phenotypic manifestations depending mainly on the amount of genetic material lost.

On the other hand, telomeric associations observed in our patient could be true chromosomal translocations rendering dicentric chromosomes and could be interpreted as an indicator of telomere shortening, phenomenon that has been directly associated with genome instability in CLL [9–11]. It is well known that during cell division, dicentric chromosomes can be pulled apart towards opposite spindle poles causing an abortion of cytokinesis, which in turn, produces both binucleated cells and NPB [5, 31, 33] as was observed in our patient (Fig. 1 c-g).

In conclusion, our CLL patient showed genomic instability in conjunction with a 13q14 deletion involving the *DLEU* and *RB1* genes. Although studies of CLL patients include the determination of the 13q14 deletion, most of them focus almost exclusively on the *DLEU* genes. It would be advisable to determine the status of the *RB1* gene in those patients with *DLEU* deletion in order to determine the size of the deletion and to make a better assessment of the prognosis. In addition, more attention should also be paid to the search for biomarkers of genomic instability in patients with CLL, as it could be a more frequent phenomenon than is currently reported.

## Abbreviations

CLL: Chronic lymphocytic leukemia
FISH: Fluorescent in situ hybridization
NPB: Nucleoplasmic bridges
